# Specialties

**Published:** 1864-07

**Authors:** 


### SPECIALTIES.

The question of specialties has nearly found its level in this country, and has been settled by admitting them in the bosom of the hospitals and centres of instruction, where they can serve purposes of progress and education within salutary limits and subject to the regulations of the general body. Left to themselves they grow rank and overrun the place in lawless outgrowths. In America, the professors of specialities have adopted the fashion of advertising. Thus we read that "Dr. Elsberg, Lecturer on the Laryngoscope and Diseases of the Larynx and Throat in the University of New York, devotes himself specially to the treatment of disease of the larynx and neighboring organs—office hours from four to six P.M.;" which announcement, with others similar to it, appears in large capitals, variously spaced, in the advertising columns of the principal weekly periodicals of America. Here there could not be any difference of opinion about the exceedingly gross impropriety of such a proceeding. However, various standards rule in different counries, and possibly the American profession may find as much reason to wonder at irregularities that we tolerate, as we do at the lax proceedings which their professional code admits.

The extent to which this system of open puffing has reached in the advertising columns of the jouraals, has led, however, to the following result. At a regular meeting of the New York County Medical Society, held on the 4th of January, 1864, the subjoined resolution was passed:—

"Resolved,—That in view of the unsettled state of opinion amongst medical practitioners concerning the propriety of advertising 'specialties' in medical and other journals, the delegates of this Society be instructed to bring this subject before the Medical Society of the State of New York at its next meeting, with the view to the establishment of some definite regulations concerning it."

In consequence of which a committee was appointed to prepare a report on the subject by the State Society, and the committee, which included the honored name of Dr. Brinsmade, on a subsequent day presented the following report and resolutions:—

"The undersigned appointed a special committee to report upon a resolution passed by the Medical Society of the County of New York in relation to the propriety of medical practitioners advertising their 'specialties' in medical or other journals, and referred to this Society for decision, beg leave to offer the following resolutions:—

"Resolved,—That in the opinion of this Society it is impossible to define the limits of advertising 'medical specialties,' either in medical or other journals.

"Resolved, — That advertisements indicating location and residence are the utmost limits of self-announcement consistent with professional dignity; and that all references to special branches of medical practice, as extra inducements to patronage, should be deemed violations of the code of medical ethics.

"Resolved,—That hereafter any medical practitioner so offending shall be deemed disqualified as a delegate to, or for membership of, this Society; and if already a delegate to or member thereof, shall be deemed a fit subject for discipline.

"Resolved,—That this Society recommends all medical societies in the State of New York to adopt the foregoing resolutions to establish the true dignity of our profession.

"Resolved,—That the foregoing resolutions be transmitted to the American Medical Association at its next annual meeting as an expression of the opinion of the medical societies of the

State of New York, and that for this purpose a Committee of presentation be appointed.

("Signed) THOMAS C. BRINSMADE,
HOWARD TOWNSAND,
GUIDO FURMAN."

The report was accepted; and, on the motion of Dr. Jenkins, the subject was made the special order for the second day of the next annual meeting.

Thus the specialists receive a check; but the admission is made that advertisements indicating location and residence are consistent with professional dignity—a proposition which it seems to us very difficult to maintain, and which would assuredly be rejected with unanimity by any English Society. So far as they go, these resolutions are of good effect; but we could desire, in the common interests of professional dignity, that they should go further.—*London Lancet.*

